# 

**Published:** 1883-12

**Authors:** 


### CONTENTS.

COMMUNICATIONS.

Replantation.......... 565
Color Blindness.......... 574
Addenda to the Report of a Case of Scurvy.......... 583
A Historical Poem.......... 592

PROCEEDINGS.

Proceedings of the Ohio State Dental Society.......... 596

EDITORIAL.

Close of Volume.......... 609
Mutual Benefit Association.......... 610
Personal.......... 611
Obituary.......... 612

